# Supplementary material for: Avialan-like brain morphology in Sinovenator (Troodontidae, Theropoda)
Source: Commun Biol. 2024 Feb 10;7:168. doi: 10.1038/s42003-024-05832-3 (PMC10858883; doi:10.1038/s42003-024-05832-3)
Supplement: Supplementary file 2 — Description of Additional Supplementary Files [file 42003_2024_5832_MOESM2_ESM.pdf]

Description of Additional Supplementary Files

File name: Supplementary Data 1
Description: Phylogenetic tree used for phylomorphospace plotting in geometric morphometric analysis.

File name: Supplementary Data 2
Description: Coordinate data of brain endocast used in this study.

File name: Supplementary Data 3
Description: 3D models of the skull, brain endocast, and endosseous labyrinth of IVPP V20378.

File name: Supplementary Data 4
Description: R code for processing and analyzing the data for this study.
